# Building an Ethical Framework for European Pharmacists in e-Pharmacies

**DOI:** 10.2196/64750

**Published:** 2025-07-04

**Authors:** Alexandra Toma, Ofelia Crişan

**Affiliations:** 1 Department of Pharmaceutical Legislation and Management Faculty of Pharmacy Iuliu Hatieganu University of Medicine and Pharmacy Cluj-Napoca Romania

**Keywords:** codes of ethics, pharmacists, medicines, e-pharmacy, ethical framework, patients’ rights, health, autonomy, justice, solidarity

## Abstract

Patients using e-pharmacies benefit substantially from their privacy and accessibility; however, they must also be aware of the health and safety risks. These risks can be mitigated through the implementation of robust legal and ethical frameworks. European pharmacists working in e-pharmacies are obligated to respect the legal frameworks governing the remote sale of medicines, as well as the rules of professional ethics, while considering the unique benefits and challenges of establishing online therapeutic relationships with patients. This Viewpoint paper aims to propose a comprehensive ethical framework for European pharmacists in e-pharmacies that promotes consistency and ensures patient rights while fostering professional integrity and responsibility. To this end, the codes of ethics of pharmacists in the European Union (EU) member states were explored to determine the extent to which they address e-pharmacies and to begin from their provisions the building of a shared ethical framework, supported by previously published findings. The ethical guidance provided by professional associations and other competent authorities of European pharmacists varies significantly across member states. In most EU member states, pharmacists’ codes of ethics do not contain rules addressing e-pharmacies. Only 8 EU member states have adopted rules guiding pharmacists’ conduct in e-pharmacies, providing detailed regulations aimed at safeguarding patients’ rights during the remote supply of medicines, health care products, and services. Considering these findings, the development of a common ethical framework for European pharmacists operating in e-pharmacies would be helpful for the development of ethics in their conduct on the internet. On the basis of the fundamental principles of biomedical ethics, we engaged in a reiterative process of reflection and discussion to develop an ethical framework tailored to the e-pharmacy context as our main contribution to knowledge. The proposed framework—representing a novel contribution to e-pharmacy ethics research—highlights ethical issues that should be incorporated into pharmacists’ codes of ethics or dedicated guidelines. These issues range from ensuring patient autonomy in selecting e-pharmacies to showing solidarity in the global e-pharmacy environment. Its novelty could be a major input for advancing e-pharmacy ethics. The following key messages are intended for pharmacists and their professional associations or other competent authorities interested in this field. Pharmacists’ codes of ethics should keep pace with the development of e-pharmacies to ensure they remain relevant and effective in guiding ethical conduct and decision-making in such an environment. Establishing a comprehensive, shared ethical framework for European pharmacists in e-pharmacies can foster a deeper understanding and appreciation of the opportunities afforded by emerging technologies. By facilitating the development of ethical conduct of pharmacists in e-pharmacies, such a framework can enhance patients’ well-being while promoting fundamental principles of autonomy, equity, and solidarity in this evolving field.

## Introduction

Since its inception, internet pharmacies have garnered support from patients, primarily owing to their more affordable prices and perceived ethical legitimacy; however, critics have raised concerns about the ethical issues related to prescribing practices within the initial business model [1]. Over time, as this business model has evolved, additional advantages for patients have been recognized, such as greater autonomy, privacy, and convenience of purchasing medicines online; improved personalization and effectiveness of patient care, the 24-hour access to various medicines and related information, including those unavailable on the local market; and safe access to medicines in a public health emergency context, such as in a pandemic [2-9]. However, some disadvantages have also been identified, including safety and security risks for patients using online pharmacies, particularly illegal pharmacies. These risks are manifold, including the lack of transparency and personal data breaches; easy access to dangerous products; encouragement of self-diagnosis; self-medication; and misuse of medicines such as antibiotics, opioids, or neuropsychiatric medicines for off-label nontherapeutic use, often maximizing the associated iatrogenic risks [2,3,5-15]. Such practices pose significant dangers when physicians do not assess patients, pharmacists do not provide professional counseling or therapy management, or inappropriate health assessments are conducted before prescribing or recommending medications [2,3,5,7,9-13].

To protect patients’ trust and safety, access to health education is crucial. Patients must be equipped to recognize and avoid unethical consultations and the purchase of unnecessary medicines or other health products. Patients also need correct and sufficient information on the legality and practices of online pharmacies and authentic medicines available online to prevent them from purchasing unauthorized, substandard, or falsified medicines from illegal websites [2-4,6,10]. Therefore, the intervention of competent authorities is essential in ensuring patient access to authentic medicines through online platforms. Many studies have emphasized the need for robust, enforceable regulations and guidelines at both national and international levels, which should be periodically updated to keep pace with technological advancements [2,4-8,10-13,16-20].

In the European Union (EU), Directive 2011/62/EU established a legal framework aimed at preventing falsified medicines from entering the legal supply chain and the need for pharmacies to be authorized by the national competent authority designated in each member state, according to the national law, to be able to sell medicines via the internet. The directive mandates that online pharmacies display their legal information, such as contact details of the national competent authority, hyperlink to its website, and the specific European logo, allowing patients to verify a website’s legality by linking it to the national authority’s list of authorized online pharmacies. The list is included in a publicly accessible register, which is essential for verifying the legitimacy of online pharmacies [21]. The directive’s provisions have been further supplemented by the Commission Delegated Regulation (EU) 2016/161, which specified the safety features appearing in the packaging of medicines for human use [22], and by the Commission Implementing Regulation (EU) 699/2014, which established the design of the European logo to identify legitimate online pharmacies and the technical, electronic, and cryptographic requirements for verifying its authenticity [23]. This legal framework is mandatory for all EU member states, which must ensure enforcement and conduct information campaigns to educate the public on the risks associated with the use of falsified medicines, prevent these risks by avoiding the purchase of medicines from illegal websites, and the role of the common European logo on legal websites [24]. In addition, the Regulation (EU) 2016/679 on the protection of natural persons regarding the processing of personal data and the free movement of such data, referred to as the General Data Protection Regulation [25], applies to these countries.

Pharmacists have a vested interest in the development of online pharmacies as a business opportunity. Simultaneously, pharmacists must remain aware of the importance of compliance with the law and professional ethics in this context [3,14,17,18], which represent a shared foundation of values upheld by professional associations. These common values foster unity among pharmacists, even in the face of the diverse sociocultural and historical contexts across different countries [26]. To be successful and remain competitive with large e-pharmacy companies, enhancing their operational efficiency and risk management strategies while managing a high volume of requests [27], independent pharmacies need to evolve. They need to integrate their online platforms with their physical locations, boost visibility on social media, and tailor their online services to meet the specific needs of their patients [17,18,28]. In this context, training pharmacists in eHealth practices is particularly critical, as emphasized by the Pharmaceutical Group of the European Union (PGEU) and the International Pharmaceutical Federation (FIP) [29-33].

The rapid advancement of digital technologies has led to the rapid evolution of eHealth practices in various fields of health care. These included telehealth, digital mental health, electronic prescribing and dispensing, integration of the Internet of Things in remote patient monitoring, use of artificial intelligence (AI) in process automation, and other specific activities of health institutions [8,34-36]. The evolving role of health care professionals, including pharmacists, is to use these technologies to service patients for better accessibility, efficiency, and safety of the necessary health products and care while maintaining their ethical responsibilities [34-38].

One key area where digital technology intersects with pharmacy is e-pharmacy, which refers to websites that provide medicines or other health products with information on their use, advice, and other specialized health services on a global scale [2,3,17,33,39]. In most countries, the ethical framework for pharmacy activities is generally adopted by professional associations of pharmacists or other competent regulatory authorities. To the best of our knowledge, a common ethical framework for pharmacists’ work in e-pharmacies has not been developed so far, although the importance of respecting ethical principles in this field has been recognized by many authors [1-5,12,14,16-18,28]. These calls for action are also supported by organizations such as the PGEU [30,32] and FIP [29,31,33].

This study aims to contribute to the development of a shared ethical framework at the EU level. Our previous studies advocated the necessity of including rules of conduct related to e-pharmacies in pharmacists’ code of ethics [40,41]. Such an approach would guide ethical conduct and decision-making in e-pharmacies, facilitate the provision of pharmaceutical products and services via the internet, and protect patients from the risks associated with purchasing medicines from unverified websites. Pursuing this endeavor in the context of the increase in the online trade in medicines, this viewpoint paper aims to propose a comprehensive ethical framework for European pharmacists in e-pharmacies that promotes consistency and ensures patient rights while fostering professional integrity and responsibility. To this end, the codes of ethics of pharmacists in the EU member states were explored to determine the extent to which they address e-pharmacies and to begin from their provisions the building of a shared ethical framework supported by previously published findings. The ethical framework for European pharmacists in e-pharmacies was proposed based on the fundamental principles of biomedical ethics [42], considering the ethical issues addressed in European pharmacists’ codes of ethics and our proposals. This approach is unique in its correlation with the ethical codes across European countries. It aims to provide practitioners from different countries with novel insights and perspectives and establishes a common foundation for e-pharmacy ethics.

## Including e-Pharmacy Provisions in the Codes of Ethics of European Pharmacists

The codes of ethics for pharmacists in the EU member states were identified and collected through documentary research [43] conducted through internet searches on the websites of national professional associations of pharmacists and regulatory authorities. We identified those entities between March and May 2023 as part of our first study within the research project on pharmacy ethics. Specifically, by searching the member organizations pages on the PGEU and FIP websites, we identified several professional associations of pharmacists, unions, and associations of pharmacies in EU countries. From these, and by checking their attributions on their own websites, we selected and included in the study the professional association responsible for adopting and implementing the code of ethics for pharmacists in each country. We searched and collected the respective codes of ethics from their websites. The default translation provided by Google Chrome was used to search for websites without official English or French versions. For failed searches, the contact person mentioned on the websites was contacted via email. In the case of the countries where we did not find a professional association responsible for adopting the pharmacists’ code of ethics, we searched the internet, using the Google search engine, to find the national regulatory authority and the respective code [44]. Between March and May 2024, and again in February 2025, updated versions of the codes of ethics for pharmacists were searched for to analyze them for this study. On these occasions, we searched for and collected other documents relevant to e-pharmacy ethics from the accessed websites.

Documentary research revealed that the Danish Pharmacists’ Association, Pharmadanmark, does not have a code of ethics for pharmacists (Thøgersen P, personal communication, May 3, 2024). The absence of a code of ethics for pharmacists in Denmark may be because the profession, including online pharmacy practice, is regulated solely through tasks outlined by the Pharmacy Act, which does not mandate adherence to such a code [45,46]. Therefore, professional associations may not have considered adopting one necessary. However, the Danish Medicine Agency requires ethical conduct in pharmacy practice, stating that “pharmacy staff must carefully and conscientiously fill and verify prescriptions as well as dispense, sell and provide information about medicines” [45]. This requirement aligns with the suggestions of Danish authors who investigated, through video-recorded meetings, pharmacists’ communication with patients during medication dispensing, recommending a more patient-centered approach aimed at evaluating options, choosing, and providing the most beneficial treatments [47]. This approach, including its application in e-pharmacies, reflects one of the fundamental principles of biomedical ethics, that of beneficence, which we have considered in the proposed ethical framework.

The conduct of pharmacists is subject to codes of professional ethics in all other EU member states. These codes of ethics are adopted and implemented by the professional association of pharmacists in 22 countries [48-69] and by the national competent regulatory authority in Ireland [70], Luxembourg [71], Malta [72], and Slovakia [73]. For this paper, all these codes were identified and collected from their websites. The default translation provided by Google Chrome was used to search for websites without official English or French versions. For codes and other relevant documents collected in PDF in languages other than English or French, their English translations obtained using Google Translate [74], DocTranslator [75], and DeepL [76] were used in the analysis process. Google Translate was used as the primary tool; however, it was unable to translate the scanned PDF files; therefore, DocTranslator, which offers quick conversion into Word format through a partner site, was used to translate these documents. These machine translation tools have substantial advantages such as free, online availability and rapid translation of different document types; however, they also have limitations related to the format of the text, the use of specialized terms or expressions, and the context in question. Meaningless expressions in documents translated from Dutch, German, and Lithuanian were mitigated by parallel translations using all 3 tools and crosschecking the results. In addition, the obtained versions were revised to ensure that the text made sense and that the message was received correctly, based on our knowledge as specialists in the pharmaceutical field. Scientists have investigated the issue of using translations in scientific literature and have recommended that specialized authors check, review, and edit the translations they use for accuracy and to overcome errors and ambiguities in machine translation tools [77].

After the necessary translations were made, pharmacists’ codes of ethics and the other relevant documents were submitted to thematic analysis to determine whether they contained provisions related to e-pharmacies as the main theme of this study and, if so, to analyze the specific issues addressed. The thematic analysis included the following steps: (1) exploring the pharmacists’ codes of ethics and other documents to elucidate the flow of the text and to become familiar with it; (2) searching for provisions related to e-pharmacies using keywords such as *e-pharmacy*, *website*, *internet*, *online*, *distance*, and *remote*; (3) identifying the issues they referred to through an inductive process of coding; (4) classifying them through a reflexive process of developing themes as shared meaning-based patterns; and (5) reviewing, interpreting, summarizing, and correlating them across countries to specify the concepts emerging from our findings, which led, in the end, to (6) building an ethical framework as a conceptual model for European pharmacists in e-pharmacies [43,78,79].

The analysis process revealed that the codes of ethics for pharmacists adopted by professional associations or competent authorities in Bulgaria [48], Croatia [49], Czechia [50], Cyprus [51], Estonia [52], Finland [53], Greece [54], Hungary [55], Ireland [70], Latvia [56], Lithuania [57], Malta [72], Poland [58], Portugal [59], Romania [60], Slovakia [73], Slovenia [61], and Sweden [62] do not contain provisions on e-pharmacies. The absence of ethical rules for e-pharmacies in those codes of ethics may indicate that those countries have not enforced the necessary legislation, regulations, and administrative measures at the national level to transpose EU legislation regarding the sale of medicines via the internet. However, this is not the case, as all member states have adopted national transposition provisions published on the EU website [80], confirming that the sale of medicines online is subject to national law in these countries. More specifically, the Directive 2011/62/EU established common safety standards for online sales of medicines, but each EU member state may impose restrictions justified by the protection of public health upon the retail supply of medicines via the internet, particularly prescription medicines, without unduly limiting the functioning of the internal market [21]. The decision of some national professional associations to not include specific rules regarding e-pharmacies in their codes of ethics for pharmacists may change in the future in response to the growing online medicine trade, especially if a common ethical framework is adopted at the European level, such as the one we propose. This finding emphasizes the significance of this study. On the other hand, e-pharmacy ethics are addressed in pharmacists’ codes of ethics, documents with explanations and commentaries on these codes, and other guidance documents used by professional associations or competent authorities at the national level in Austria [63], Belgium [64], France [65,81-83], Germany [84,85], Italy [67], Luxembourg [71], the Netherlands [68,86,87], and Spain [69,88]. Table 1 presents an illustration of these findings according to country.

**Table 1:** Documents addressing ethics in e-pharmacies in European countries.

Countries	Code of ethics	Code of ethics commented	Other guiding documents
Austria		✓	
Belgium	✓	✓	
France		✓	✓
Germany			✓
Italy	✓	✓	
Luxembourg	✓		
Netherlands			✓
Spain	✓		✓

The extent to which e-pharmacy ethics are addressed in these documents was analyzed through an inductive and reflexive process of coding and developing themes. This process led to the identification of specific elements of e-pharmacy ethics expressed as codes and their classification into 3 main themes as shared meaning-based patterns. Therefore, they reflect not only the content of the provisions of the pharmacists’ codes of ethics and the other documents analyzed but also our own understanding and interpretation based on our experience and expertise as pharmacists and scientists. These findings reveal that the coverage of e-pharmacies varies significantly, with specific issues being explored in greater or lesser detail depending on the country. In particular, they relate to website design and content, pharmacists’ online relationships and communication with patients, online advertising, and pharmacy business practices. Table 2 summarizes the findings according to country.

**Table 2:** Ethical issues of pharmacists in e-pharmacies addressed in European countries.

Country	Themes and codes		
	Website design and content	Professional relationships and communication with patients	Advertising and business practices
Austria [63]	Legal content Accurate content	Providing clear information and free counseling Documenting counseling Ensuring medication safety Encouraging rational purchasing	Fair promotion of medicine delivery Fair competition and advertising
Belgium [64]	Robust design Legal content Accurate content Head pharmacist’s responsibility	Professional transparency Therapeutic relationship or access to information to ensure quality and reduce risks Respecting patients’ privacy Protecting confidentiality Professional independence Documenting counseling Obtaining free and informed consent for specific interventions Encouraging rational purchasing Ensuring the safety and quality of health product delivery Providing a complaint form Maintaining patient trust	Fair competition and advertising Head pharmacist’s responsibility
France [81-83]	Legal content	Respecting patients’ privacy Protecting confidentiality Obtaining free and informed consent for specific interventions Ensuring medication safety Maintaining patient trust	Fair competition and advertising
Germany [84,85]	Secure eHealth environment	Supporting patients in eHealth Equitable access to health care Solidarity, avoiding exclusion Focus on patients’ well-being Improving quality and results Assessing costs, benefits, and risks Respecting patients’ privacy Protecting confidentiality Maintaining patient trust Interdisciplinary collaboration in the patients’ best interest Transparency in using AI^a^ Professional independence in decision-making supported by AI Improving advice through AI	Fair competition between eHealth and traditional health care practices Legal and ethical use of AI in business practices
Italy [67]	Legal content	Acting in the patient’s best interests as health care providers Professional independence	Fair competition and advertising
Luxembourg [71]	Robust design Legal content Accurate content Head pharmacist’s responsibility	Professional transparency Therapeutic relationship or access to information to ensure quality and reduce risks Respecting patients’ privacy Protecting confidentiality Obtaining free and informed consent for specific interventions	Fair competition and advertising Head pharmacist’s responsibility
Netherlands [86,87]	Legal content Accurate content	Professional transparency Therapeutic relationship or access to information to ensure quality and reduce risks Ensuring medication safety Respecting patients’ privacy Protecting confidentiality Professional independence Maintaining patient trust Avoiding conflicts of interests Recommending reliable sources of information to patients Encouraging rational purchasing Ensuring safety and quality of health product delivery Providing a complaint procedure	Fair competition and advertising
Spain [69,88]	Legal content Accurate content	Professional transparency Therapeutic relationship or access to information to ensure quality and reduce risks Ensuring medication safety Respecting patients’ privacy Protecting confidentiality Obtaining free and informed consent for specific interventions Maintaining patient trust Interdisciplinary collaboration in the patients’ best interest Transparency in using AI Community sustainability	Fair competition and advertising

^a^AI: artificial intelligence.

The professional associations in these 8 countries appear to recognize that the novelty and challenges of professional communication on the internet may lead to difficulties in applying ethical principles in online interactions with patients. Therefore, they have outlined appropriate conduct to address issues and key ethical concerns in this context.

In Austria, the rules of pharmaceutical ethics are detailed in the professional code of conduct adopted by the Austrian Chamber of Pharmacists. Although the code does not specifically address e-pharmacies, it allows for advertising on pharmacy websites and extensively addresses competition and advertising issues. Each section of the code includes commentary on the relationship between applicable legislation and the practical application of the ethical rules of that section [63]. e-Pharmacy activities are specifically addressed in numerous comments, providing an overview of their most important ethical aspects for Austrian pharmacists. Thus, the pharmacy website must have legal content provided by national legislation and regulations, including clear and concise information about medicines, their correct and safe use, and consultation with the pharmacy if clarification is required. It must have a distinguished design that aligns with the status of the pharmacy as a health institution and must have accurate and nonmisleading content. Pharmacists must provide the patient with information and advice for the correct and safe use of medicines on the pharmacy’s website and via telephone or email. They must document pharmaceutical advice and send medicines to patients only after having clarified any uncertainty, avoiding the dispatch of products in case of “justified suspicion of misuse, e.g., when ordering large quantities of a medicinal product with potential for misuse” [63]. If registration as a distance-selling pharmacy exists, pharmacists should only use legal methods and materials for advertising, such as the pharmacy’s website or other retail portals approved by the Austrian Chamber of Pharmacists. Pharmacists are required to avoid illegal advertising, such as encouraging the internet purchase of prescription medicines, promoting their delivery service without the chamber approving such service, and promoting the delivery of over-the-counter medicines without distance-selling pharmacy registration [63].

In Belgium, the Code of Pharmaceutical Deontology adopted by the Order of Pharmacists contains a section on the particular duties of pharmacists in e-pharmacies [64]. In addition, the code refers to a communication titled “Online Pharmacy Activities. Deontological Aspects,” which is included in the commented code and provides important specifications on the application in practice and examples of cases of violation of provisions addressing e-pharmacies [64]. The code requirements underline the great interest of Belgian pharmacists in the ethics of e-pharmacies. Thus, the head pharmacist is responsible for ensuring compliance with the law of the website and its content, including interactive and individualized communication with patients. The website must comply with ethical standards in online activity, and it must be considered “a virtual extension” of the community pharmacy [64]. Clarity, neutrality, and sobriety are required for the presentation on the website and the health products sold through it. Transparency is required from the professional in the pharmacy team (eg, name and position) who receives and processes the patient’s request. Protecting professional secrecy and patient privacy by adequately securing information that is exchanged online is mandatory. The commented code specifies that the existence of a prior therapeutic relationship between the pharmacist and the patient placing the online request or obtaining sufficient information about that patient is necessary to enable the pharmacist to provide quality pharmaceutical services and minimize the risks associated with the requested health products. Documenting the exchange of information with the patient is required, either in the pharmaceutical file, with the patient’s consent, or through printing a discussion with the pharmacist. The complaint form used in the physical pharmacy must be available to the patient online so that they can express any dissatisfaction related to the services provided in e-pharmacies in a standardized way. Pharmacists should ensure the quality and safety of the delivery of medicines and other health products to patients requesting e-pharmacy services [64]. Promoting the rational use of medicines includes limiting the quantities sold online to those necessary for treatment based on scientific evidence, with no minimum quantity imposed and no other products sold for purely commercial purposes, as mentioned in the commented code. Maintaining professional independence and proving dignity, discretion, and restraint in advertising and commercial practices are required to preserve pharmacists’ credibility and probity and their relationship of trust with the patient [64]. The Belgian Order of Pharmacists addresses e-pharmacy ethics in great detail, reflecting a strong stance aimed at preventing the profession from succumbing to commercialism and preserving its nobility, even in the online domain.

In contrast, France’s Code of Deontology, adopted by the National Order of Pharmacists [65], does not include any specific provisions for e-pharmacies. However, the commented Code of Deontology references instances of ethical misconduct by pharmacists related to internet use in professional activities [81]. The French National Order of Pharmacists places significant importance on professionals and has issued a specialized guide titled *Recommendations for Respecting the Confidentiality of Patient Data in the Use of Information Technology*. Although most recommendations focus on the administrative and technical aspects of ensuring the security of personal data, they also integrate elements of professional ethics [82]. From the documents of the French National Order of Pharmacists, the most important ethical issues relevant to e-pharmacies have been identified. The legality of the content of the pharmacy website involved in the sale of medicines via the internet is promoted by the French National Order of Pharmacists as a competent authority [83]. Pharmacists should respect patients’ rights to privacy and the protection of personal information, be informed about information collection and processing, object to such actions, have access to all personal data, and request their rectification, including erasure. They should assume correlative duties to maintain professional secrecy, comply with technical and organizational obligations to ensure the security of information systems, and seek the patient’s free and informed consent for the collection and processing of personal data, for example, for the pharmacist’s compilation and completion of the patient’s pharmaceutical file [82]. In addition, pharmacists should ensure complete patient identification and advice when selling medicines over the internet, especially if only telephone and email are used for communication to provide the necessary framework for patient counseling on medication safety. They should avoid unfair competition by offering patients gifts imprinted with the pharmacy’s website address and unethical advertising on the internet by associating the pharmacy’s logo with the contact details of the insurer [81]. These duties are integral to good pharmacy practices applicable to the electronic trade of medicines [89]. Access to the pharmaceutical file maintained by French pharmacists, containing a patient’s recent medication history, is particularly useful for the e-pharmacy dispensing of medicines. This tool promotes the continuity and safety of pharmaceutical services by aiding in the identification and management of risks such as redundant treatments, medication errors, and interactions [81]. Considering the French National Order of Pharmacists’s focus on improving pharmacists’ conduct, future revisions of the Code of Deontology will address e-pharmacy ethics issues.

In Germany, no provisions specific to e-pharmacies exist in the professional codes of conduct of the 17 chambers of pharmacists and members of the Federal Chamber of Pharmacists [66]. Notably, the Bremen Chamber of Pharmacists recognizes in its code the competence of pharmacists to practice in digitalization, especially regarding the management of information associated with medicines and their prescription and dispensing to patients [90]. In contrast, the Federal Union of German Associations of Pharmacists (Bundesvereinigung Deutscher Apothekerverbände [ABDA]), comprising all state chambers and associations of pharmacists in Germany [91], has adopted a position paper titled “E-health: Ethical Principles,” to guide pharmacists in maintaining ethical values in the digitization of the health care system [92]. The most relevant principles from the ABDA guidelines maintain that creating a secure e-pharmacy environment requires close collaboration between specialists from various fields to ensure the existence and protection of the IT infrastructure and processed data, as well as the implementation of a framework of legal and ethical standards to be adhered to throughout their use. Pharmacists should focus on the well-being and safety of patients and the use of new technologies to optimize the quality, effectiveness, and efficiency of pharmaceutical services. They should choose the best way to communicate and interact with patients to support them in managing their health problems using new technologies while being aware of the importance of maintaining patient trust as the basis for a professional relationship, even in an online environment. In the use of these technologies, the principles of pharmacoeconomic and societal interests should be considered. Cooperation with other professionals in the eHealth network, multidisciplinary teams working in the best interests of patients, and fair remuneration for professional services are necessary. Ensuring fair and nondiscriminatory access to e-pharmacy services, showing solidarity, and preventing digital exclusion are required. Proper protection of patient health data and their use only for legitimate purposes are mandatory. In addition, pharmacists should use fair practices in the competition between e-pharmacies and traditional pharmacies while improving patients’ possibilities of accessing medicines and services necessary to achieve their health goals [84]. Recently, ABDA introduced another position paper titled “Use of Artificial Intelligence (AI) in Pharmacy,” which emphasizes the importance of adhering to legal and ethical standards in the application of AI technologies. This document highlights critical principles, including transparency, data security, and quality assurance. Pharmacists are advised to “use it as a support, not as a replacement,” while remaining responsible for their professional relationship with patients [85]. The study and reflection on these principles highlight a policy framework at the organizational level, demonstrating ABDA’s commitment to the necessity of an ethical framework for e-pharmacies. This stance supports the broader call for a unified ethical framework for European pharmacists.

In Italy, the Federation of the Order of Italian Pharmacists has published the Code of Deontology accompanied by a commented version [67]. Both versions are concise regarding e-pharmacy activities, referring to core principles and summarizing professional attitudes pharmacists must uphold in their online practices. Thus, the website must comply with the technical requirements and regulations indicated in the commented-on code, along with legal sanctions applicable by the authorities in cases of noncompliance, including the closure of illegal websites. Pharmacists are obligated to act independently in the interest of patients’ health by respecting general ethical principles. They must protect the image and reputation of their profession, including in online trading practices [67].

In Luxembourg, the Pharmacists’ Code of Deontology, enacted by the medical college, contains various provisions related to e-pharmacies. The most interesting ones specific to Luxembourgish pharmacists state that the pharmacy website is “a public health space” [71]. The head pharmacist is responsible for website legality, security, and content, including the links it may contain, and must seek the medical college’s agreement. The presentation of pharmaceutical activity on the website must be clear, neutral, and objective. Pharmacists may accept requests for medicines on the website; however, medicines may only be dispensed at the physical pharmacy, even if the patient’s anamnesis and counseling have been conducted online, respecting their rights to privacy and confidentiality. Also, pharmacists must ensure an interactive communication system with patients that guarantees informed consent for the sale of other health products via the internet. They have no right to use any information they have access to for commercial purposes by virtue of their online therapeutic relationships with patients. Pharmacy websites should not be used as tools for unethical advertising, and online practices to attract customers or requests are prohibited [71]. The prohibition of distance delivery of medicines in Luxembourg stands out, with the regulatory authority stressing the pharmacist’s responsibility to ensure appropriate storage conditions, quality assurance, and maintenance of their therapeutic properties.

In the Netherlands, the Royal Dutch Pharmacists Association (Koninklijke Nederlandse Maatschappij ter bevordering der Pharmacie [KNMP]) includes principles and rules of pharmaceutical ethics in several documents, such as the Code of Conduct [68], Professional Statute [93], and Charter Professionalism of the Pharmacist [94]. However, these do not specifically address e-pharmacies. Instead, e-pharmacy is covered in the KNMP Guidelines Practice Management. These guidelines are formulated primarily as quality standards for pharmacists’ online activities; however, some also contain essential professional ethical requirements [86] or refer to such requirements in the Code of Conduct for Pharmaceutical Advertising [87]. The pharmacy website should contain all information regarding the online provision of pharmaceutical products and services, including a complaint procedure, so the patient can make an informed decision when purchasing medicines through e-pharmacies. The suitability of the form of remote communication with the patient is indicated, including the use of video means, for pharmacists to receive feedback through nonlinguistic interaction. Pharmacists should respect privacy legislation in situations where the patient’s data and information, including health information, must be requested or provided to another pharmacy or health facility. They should maintain professional independence to preserve patients’ trust, which is the basis of their therapeutic relationship, along with knowledge of their medical and medication history. They should ensure the independence and accuracy of online sources of information recommended to patients without encouraging the purchase of products and services over the internet. They should guarantee the delivery of medicines directly to the patient or their representative while respecting quality and safety [86]. Pharmacists should use sustainable business operations and responsible, competitive practices [86,87]. The thorough and complex content of professional documents produced by the KNMP is a permanent source of inspiration and reflection for developing quality and ethical standards for pharmacists, including those working in e-pharmacies.

In Spain, the Code of Deontology of the Pharmaceutical Profession, approved by the General Assembly of Official Colleges of Pharmacists, contains a chapter on professional requirements related to internet communication and the provision of remote pharmaceutical services. The requirements that are different from those previously mentioned stand out through a particular wording style to ensure the delivery of the correct message. For example, the pharmacist “must ensure that the information they issue is comprehensible, truthful, well-considered, prudent, simple and in accordance with the law and appropriate to the needs of the forum in which it is issued” [69]. In an e-pharmacy, the pharmacist should be aware that “the ethical responsibilities existing in face-to-face performance must be maintained, with special emphasis on reinforcing the values of transparency, information, trust, and confidentiality, complying in all cases with the current legislation on the matter” [69]. In any communication on the internet, including with their competitors, pharmacists “must always avoid personal disqualification and insults, as well as scandal and public discredit of the profession” [69]. The values and virtues of pharmacists in e-pharmacies are fully supported, following the ongoing professional development work of the Spanish association with great interest. Moreover, the association has adopted a “digital agenda” for pharmacists to create a digital pharmaceutical network in which digital health solutions and AI technology are used responsibly and transparently. This initiative aims to include all pharmacies in Spain, ensuring the provision of pharmaceutical products and services at the local community level in an environmentally sustainable manner while upholding quality and professional, ethical standards, such as transparency, prior consent, privacy, confidentiality, medication safety, and interdisciplinary cooperation for the benefit of patients [88]. These goals of the Spanish association, particularly the increased interest in keeping pharmacists close to patients and the community—including through e-pharmacies—while contributing to environmental protection, align with our position on current professional priorities.

It is worth specifying that all provisions in the codes of ethics are binding for pharmacists and professional associations, setting clear rules for ethical conduct. For instance, in Belgium and France, commented codes outline instances where pharmacists have faced sanctions for failing to comply with provisions of their codes of ethics [64,81]. Although other documents, such as comments, recommendations, position papers, guidelines, and digital agendas, are not binding, they reflect the views of professional associations and may influence future amendments to codes of ethics. In some cases, the codes of ethics reiterate or extend the principles established by legal texts, as is the case with patients’ rights. These are typically well encapsulated in the legislation of EU member states, as the European Commission has shown in a report on this subject; however, they may face challenges in practical implementation [95]. The reiteration of these principles in the codes of ethics reaffirms the commitment of associations to fostering good conduct in pharmacist-patient relationships, including in e-pharmacy activities.

The literature highlights critical standards that pharmacists must adhere to in e-pharmacy practices, such as displaying ethical policies in relation to patients on the website [2,14]; ensuring integrity in e-commerce with medicines and promoting it ethically [4]; developing patient education and awareness to recognize illegal e-pharmacies [5,10,11], including through the use of web analytics [6]; or collaborating in preventing supply disruption to avoid the use of fake medicines purchased from illicit online sources [12]. Numerous authors stress that competent authorities should enact programs [2,8], develop guidelines [7,11,13], and launch campaigns [4,12,17] to mitigate the risks associated with abuse [10], falsified medicines [10,12], and their environmental impact [4] while ensuring equitable [13] and safe access to quality medications and pharmaceutical services [2,4,5,8,10-17]. Using new technologies [19,20], they could develop a national and international consensus to adequately protect the safety of patients purchasing medicines online [5,12]. Bessell et al [2] noted that “the challenge is to discourage fraudulent and misleading websites but permit the development of innovative, ethical e-pharmacy services.” This perspective aligns with the current proposal to include targeted e-pharmacy provisions in the ethical codes of pharmacists or professional guiding documents. Moreover, by designing the ethical framework by correlating the codes of ethics of European pharmacists, this study offers a novel approach to e-pharmacy ethics, thereby providing practitioners with insights and perspectives on the preoccupations and contributions of their peers from other countries, which can enrich their practices and patients’ interests.

This rapid expansion, fueled by digital advancements and pharmacist education, increasingly incorporates hybrid models combining theory with hands-on training in digital contexts [8]. Internationally, the FIP recommends educational programs for pharmacists that include knowledge, skills, and attitudes related to substandard and falsified medicines to help patients safely obtain medications via the internet while adhering to professional ethics [29]. In addition, the FIP anticipates that “the implementation of more digitization will bring a magnitude of ethical and philosophical questions on privacy, on autonomy, on data-sharing responsibilities and requirements, and on accessibility and solidarity” [31]. These ethical issues are important and were thus addressed in this study.

The PGEU states that pharmacists should adhere to the same rules of professional ethics, whether they are involved in on-site or online activities that use digital technologies to provide necessary products and services to patients [32]. However, the FIP goes further by asserting that “as custodians of medication management systems, pharmacists have a responsibility to ensure that the use of such technologies is supported by strong regulatory and ethical frameworks” [33]. The proposed ethical framework strongly aligns with this objective, as we built it by considering the European regulatory framework and attempting to address the essential ethical issues that pharmacists may face in e-pharmacy activities.

## Building an Ethical Framework for European Pharmacists in e-Pharmacies

### Overview

To achieve the aim of this study, an ethical framework for European pharmacists in e-pharmacies was established based on the fundamental principles of biomedical ethics, including respect for patient autonomy, nonmaleficence, beneficence, and justice [42]. This was accomplished by reviewing, interpreting, summarizing, and correlating codes and themes across countries and building “brick by brick,” a conceptual model for e-pharmacy ethics. Given the differences in the approach to e-pharmacy ethics in European countries—ranging from the absence of specific provisions in most cases to detailed regulations through codes of ethics and other specific guiding documents—a common framework that organizes and addresses ethical issues specific to e-pharmacies is necessary and useful. Therefore, the framework was structured around the practical aspects of pharmacists’ work, from the establishment of e-pharmacies to the provision of products and services, both locally and across European borders, ensuring that essential ethical issues are addressed in line with biomedical ethics. The proposed ethical framework is presented in Table 3.

**Table 3:** Ethical framework for European pharmacists in e-Pharmacies.

Fundamental principles	Ethical issues that specific norms in codes of ethics and specific guidelines should address for pharmacists in e-pharmacies
Respect for patients’ autonomy	Acting in the patient’s best interests by giving the choice of e-pharmacy Robust website design with legal and accurate content for patients Providing clear information on accessing e-pharmacy services Professional transparency from the first online contact with the patient Providing clear information on health issues and available treatments Respecting patients’ privacy in a professional online relationship Protecting the confidentiality of patients’ information in digital format Obtaining free and informed consent for pharmaceutical interventions Helping patients make health care decisions if they ask for it online Providing clear information on medicines and other health products that can be offered, chosen, and supplied remotely
Nonmaleficence	Avoiding malpractice and errors by following the relevant procedures Avoiding technology malfunction and data security breaches Avoiding offensive and abusive online communication
Beneficence	Protecting and defending patients’ rights to health protection Providing access to health products and services through an e-pharmacy Recommending reliable online sources of information to patients Establishing therapeutic relationships with patients or asking for access to their information to provide personalized treatment in an e-pharmacy Evaluating options and providing beneficial treatments to patients Providing proper online counseling on medicines and other health products recommended, chosen, and supplied remotely Preventing or mitigating risks associated with the chosen treatments Improving the quality and results of the selected treatments Supporting community and environmental sustainability Providing pharmaceutical products and services remotely in medically deserted areas or those affected by plagues, disasters, or war
Justice	Ensuring fair, equitable, and appropriate treatment of online patients Maintaining professional independence in providing online health care Avoiding conflicts of interest in relationships with online patients Fair online advertising, clearly distinct from professional information Showing solidarity and preventing patients’ exclusion from an e-pharmacy Supporting patients in the global e-pharmacy environment Protecting patients’ rights to have fair access to and at least basic pharmaceutical care or helping them to have a fair opportunity to do so, for example, by providing them with cross-border access to e-pharmacies

In the following sections, we have explained our rationale and substantiation regarding the reflection of fundamental principles of bioethics in the framework we propose for e-pharmacies.

### Respect for Patients’ Autonomy

The first fundamental principle of biomedical ethics is respect for patient autonomy, which supports rules on veracity, privacy, confidentiality, consent for interventions, and providing help to the patient in making informed decisions [42]. Professional associations should encourage community pharmacies to organize themselves as e-pharmacies to offer this choice to all patients as an application of their right to respect for their autonomy, in line with the evolution of IT, which, according to the German position paper on ethical principles in eHealth [84] and the Italian Pharmacists’ Code of Deontology [67], should be used in the interest of patients’ health. The professional design, transparency, and accuracy of the e-pharmacy websites, emphasized by the Belgian [64] and Luxembourg [71] pharmacists’ codes of deontology, should enable patients to make informed decisions over illegal platforms. e-Pharmacy websites should present clear, reliable, and professionally vetted content, reinforcing the trust that patients place in the online services provided by competent, honest, and polite pharmacists. In addition, as a sign of respect for patients’ autonomy when accessing e-pharmacies, pharmacists should assist them in understanding their health problems, making decisions on treatments, and following prescribed treatments provided remotely. Simultaneously, the patient should be fully informed to consent to the pharmacist’s interventions, such as sharing personal health data with other professionals in the patient’s best interest. Patients should know and trust that their privacy is protected and that their data, including sensitive health-related data, are securely managed in e-pharmacies. Both the privacy of purchasing and the privacy related to the pharmaceutical services provided during the dispensing of medicines in e-pharmacies should be ensured by pharmacists, as very well exemplified in the KNMP Guidelines [86]. Respecting patients’ privacy and protecting the confidentiality of patient information are core ethical requirements shared across documents produced by professional associations. However, some associations, such as those in Austria and Italy, do not explicitly address e-pharmacies in relation to privacy. Nevertheless, all associations refer to the General Data Protection Regulation principles, which establish that patients should be informed about the processing of their data and related rights, that the collected data should be minimized and limited to the purpose, and that the accuracy and security of the collected data should be ensured, including the application of the ethical obligation of professional secrecy [25].

Respecting patient autonomy is paramount in e-pharmacies, as it facilitates patients’ rights to self-determination, including access to necessary medications. Vulnerable groups, such as older adults and uninsured individuals, particularly benefit from these developments [1]. However, autonomy must be balanced with professional oversight to prevent misuse or overuse of medications [3]. Even regarding the use of neuropsychiatric medicines as neuroenhancers by healthy patients, Vecellio Segate [15] states that “they should be those who eventually get to decide after receiving nonbinding advice in the form of guidelines and expert opinions.” Other authors also mention the dangerous implications of such use, including effects on individual autonomy and freedom [9]. Pharmacists’ responsibilities to obtain informed consent, protect patient privacy, and ensure their safety and well-being are recognized by organizations such as the PGEU [30,32] and FIP [31,33]. Therefore, patients using e-pharmacies need to have access to quality information on websites, specific education, and competent advice from pharmacists through means that allow synchronous communication [2]. In this way, pharmacists can assist patients in making informed decisions [4], as an essential element in exercising autonomy. Privacy and data confidentiality are equally important, requiring collaborative efforts between web developers, data protection officers, and pharmacists to address potential risks during website development. This includes informing patients about data collection, safeguarding sensitive health data, and preventing accidental leaks [14]. Furthermore, veracity and transparency are pivotal in all aspects of e-pharmacy activities, from the website and associated policies [2,3,8,14]—including editorial and commercial ones [16,18]—to organizing online work [7], patient reception, anamnesis, counseling [2,4], and safe management [5,8,14].

Moreover, the European Association of e-Pharmacies (EAEP), in its position paper “Principles for Ethical Use of Artificial Intelligence (AI),” expresses its support in preserving autonomy through human verification of AI systems used in e-pharmacies and for ensuring transparency, privacy, and confidentiality in their use [38]. These commitments resonate strongly with the proposed ethical framework, providing a robust foundation for the responsible evolution of e-pharmacies.

### Nonmaleficence in e-Pharmacies

The second fundamental principle of biomedical ethics is nonmaleficence, which supports rules on not killing; not producing pain, suffering, or hurt; not offending; and not depriving people of the goods of life [42]. This principle is linked to a particularly important aspect of e-pharmacies, namely quality assurance as a professional and ethical obligation of pharmacists. Quality assurance requires compliance with rules of good practice adapted to the supply of pharmaceutical products and services remotely, such as those developed in France [89]. These rules should be implemented by developing and following e-pharmacy–specific standard operating procedures in practice. Such procedures will help guide pharmacists’ decision-making [30] and avoid patient harm, including malpractice or errors [42]. For example, dispensing neuropsychiatric medicines for off-label nontherapeutic use as neuroenhancers without reliable evidence regarding their efficacy and safety should be considered malpractice. In such cases, the pharmacists would be liable, particularly if the injured patient did not have a medical prescription when accessing the e-pharmacy. Therefore, pharmacists should refuse such a request, including in e-pharmacies, and explain to the patient the risks of using those medicines outside of therapeutic indications. In managing e-pharmacy operations, including patient health and treatment information, pharmacists should be familiar with how to operate e-pharmacy IT systems, ensuring that they are properly maintained and kept up-to-date. This requirement is highlighted in the French guidelines [82], the German position paper on ethical principles in eHealth [84], and the codes of deontology of pharmacists in Luxembourg [71] and Spain [69]. These practices are meant to avoid wronging patients [42] by system malfunctions or data security breaches. Furthermore, online communications with patients must always respect their dignity, which is in line with the Spanish Code of Deontology [69].

The ethical issues in relation to nonmaleficence are supported by the positions expressed by some authors, as well as by the FIP and PGEU in their statements, which emphasize that avoiding medication errors is an essential patient safety concern in the context of digitization. Various solutions involving health IT have been identified, including enabling pharmacists to access patients’ electronic health records (EHRs) [32], advancing software development, and using robotics or AI to improve efficiency and reduce human errors [8,27], as also recognized by the FIP [31]. Preventing website vulnerabilities and data security breaches requires implementing specific analyses and measures by web and software developers, as well as data protection officers. However, pharmacists need to know that they are also responsible for these risks in their interactions with patients [14] and must monitor and resolve them efficiently with IT experts [32]. Therefore, training pharmacists in digital technologies and ensuring their ongoing access to IT expertise—including avoiding technology malfunctions and technology-induced errors—is essential, as emphasized by the FIP [31,33] and PGEU [32]. Moreover, an ethical principle from the German position paper on eHealth ethics stresses that the risks and costs associated with new technologies must undergo rigorous evaluation to ensure that they do not outweigh their benefits regarding patient and societal effectiveness and quality [84]. Regarding the use of AI in e-pharmacies, the German position paper similarly warns of the need to assess and minimize its risks [85]. Besides, the EAEP argues that not harming humans should be its main characteristic, and its adequacy and robustness must be ensured to mitigate safety and security risks [38]. In addition, avoiding iatrogenic harm stemming from commercial practices in e-pharmacies—a concern raised by other authors [3,4]—aligns with the ethical behavior promoted by pharmacists’ associations in Austria, Belgium, and the Netherlands. These organizations advocate for professionalism, caution, and a policy of refusing online requests for large quantities of medication [63,64], encouraging rational online purchasing [86].

### Beneficence in e-Pharmacies

The third fundamental principle of biomedical ethics is beneficence, which supports rules on protecting and defending patients’ rights, providing beneficial treatments, preventing patient harm, and helping people with disabilities and those in danger [42]. Pharmacists’ professional associations should support their members’ e-pharmacy activities by developing specific rules of proper conduct to respect patients and ensure that patients’ rights to health protection are respected. These rules should encompass everything from maintaining a compassionate attitude in communications with patients using online services to offering clear information and ensuring the provision of medications and health products along with necessary counseling and risk management. Pharmacists should use technologies that allow audio and video communication with patients in e-pharmacies to coordinate verbal and nonverbal messages, demonstrate medical device use if necessary, and ensure personalized discussions and understanding of the advice given, as stipulated by the codes of deontology of pharmacists in Belgium [64] and Luxembourg [71] and the KNMP Guidelines [86]. Furthermore, through these interactions, pharmacists can express compassion and support for patient’s health issues, helping them thoughtfully and with integrity. This approach strengthens patient trust and ensures the professional relationship remains personal and respectful, avoiding the depersonalization of the care process [42].

The existence of a therapeutic relationship between the pharmacist and the patient, along with access to the patient’s medical and medication history—emphasized by professional associations in Belgium [64], Luxembourg [71], the Netherlands [86], and Spain [69]—is an essential foundation for tailoring e-pharmacy services and products to individual patient needs. This relationship facilitates treatment monitoring and assessment of therapeutic outcomes. In addition, the German position paper on the use of AI in pharmacy highlights that “in order to sustainably increase behavioral changes and adherence to interventions and therapies, human-to-human contact is essential” [85]. Accordingly, the Spanish digital agenda advocates using AI to optimize administrative processes in pharmacies, enabling pharmacists to dedicate more time to patient care and monitoring medication use [88]. According to European legislation, over-the-counter medicines can be supplied remotely via e-pharmacies, which necessitates pharmacists’ obligation to be informed about the patient’s medical and medication history and the legal status of the medicines they provide to patients in different countries [21]. However, cross-border e-pharmacies can also develop toward dispensing prescription medicines. The interoperability of e-prescriptions and EHRs is expected to further expand the scope of cross-border e-pharmacies [96], facilitating access to prescription medications and addressing shortages in national markets. The proposed ethical framework remains valid for this development of e-pharmacies at the European level.

Pharmacists’ beneficial actions in e-pharmacies are not limited to ensuring the quality and safety of pharmaceutical products accessed online but also extend to contributing to the sustainability of local communities and responding to public health emergencies, as highlighted in the Spanish digital agenda [88].

The ethical issues related to the principle of beneficence are primarily supported at the international level, where accessibility, improvement of health and digital literacy, increased adherence to treatment, and optimization of disease management are highlighted among the benefits of e-services that pharmacists provide to patients, according to the PGEU [30] and FIP [31,33]. Many authors have shown that e-pharmacy activities, if well-regulated [4,8] and organized [7,18], significantly benefit patients [2,13], including vulnerable groups such as older individuals and individuals with disabilities [1,18,28]. These services can address health concerns more conveniently, ensure personalized services [8,39], and enhance the efficiency, quality, and safety of treatments [4,40,41]. In this context, the FIP and PGEU advocate for integrating EHR systems into pharmacists’ activities, “which, if implemented correctly, could bridge the information gap between health care professionals and patients” [32] and help fulfill their professional obligations and share responsibility for achieving and improving the therapeutic outcomes of medicinal treatments [30,31]. This perspective aligns with the specific requirement included in the proposed ethical framework for pharmacists, which emphasizes that access to a patient’s medical and medication history is essential to establishing a meaningful therapeutic relationship. By gaining a comprehensive understanding of patients, e-pharmacy pharmacists can also improve their quality of life and environmental sustainability (eg, delivery by bicycles) [32,33] by supplying all necessary medications and other health products to the local community, delivering them to patients’ homes, on time, and in the necessary quantities. Moreover, other authors have mentioned that pharmacists could play a crucial role in addressing health [3-5] and environmental risks [4]—such as those posed by falsified medicines purchased online—through awareness campaigns [4,5]. Notably, the EAEP has also endorsed the promotion of sustainability, including through AI systems used in e-pharmacies [38]. In the current European context complicated by pandemics, war, migration, and climate change, e-pharmacies offer various ways for pharmacists to express their commitment to social responsibility, community, and environmental sustainability.

### Justice in e-Pharmacies

The fourth fundamental principle of biomedical ethics is justice, which supports rules on fairness, equity, and appropriate treatment of patients [42]. Pharmacists’ beneficial actions and the justice principle are intrinsically linked in e-pharmacies, given the fast dissemination of information online and its potential for misuse. The Order of Pharmacists in Belgium stresses the importance of maintaining independence from external interests, avoiding exaggerated advertising, and separating professional obligations from promotional activities [64]. Avoiding conflicts of interest in e-pharmacies, for example, because of sponsorships and separating professional information from promotional information, is also supported in the Netherlands through the KNMP Guidelines in correlation with the Code of Conduct for Pharmaceutical Advertising [86,87]. Therefore, these ethical issues were included in the proposed framework. These considerations, alongside the German position paper’s call for equitable access to eHealth [84] and the emphasis on global justice by Beauchamp and Childress [42], form the basis for including cross-border solidarity within the ethical framework—an acknowledgment of the need to assist patients during local or global crises.

The ethical issues discussed under the principle of justice are supported by the positions of the FIP [31,33], and PGEU [32], and research findings highlight the need for fairness in access to e-pharmacy services [13,14]. This includes prioritizing the development of services tailored to vulnerable populations to prevent digital exclusion [1,18,28]. Any internet-based communication by pharmacists, including for advertising purposes, must adhere to ethical standards to protect patient trust in e-pharmacies [16,18]. Therefore, promotional and professional materials must be separated on pharmacy websites [16]; that is, information must be used in a responsible and nonmanipulative manner, especially in advertising [3,5]. Justice in pharmacists’ work in e-pharmacies can be manifested by providing special assistance to digitally vulnerable patients [3-5], especially regarding accessing legal e-pharmacy services, ensuring the availability of necessary products, and avoiding illegal e-pharmacies while explaining the risks and dangers they are exposed to otherwise [40,41], as also maintained by the FIP [29]. In addition, the EAEP calls for the integration of AI systems in e-pharmacies that prioritize fairness, nondiscrimination, diversity, inclusion, and accountability [38], which is subject to the commitment of all stakeholders. The FIP also stated that “digital transformation may allow for more inclusive, equitable, and ethical use of healthcare resources” [33]. Every pharmacist has the opportunity to contribute, at least through health and medicine information and advice, to create a fairer world, given that e-pharmacies are accessible to anyone with internet access. As the primary contribution of this study, the proposal of an ethical framework is essential because it is relevant from a theoretical point of view and to the real world through its potential to affect policies and practices in e-pharmacies.

This claim is reinforced by the fact that preoccupations about eHealth ethics and proposals for specific ethical frameworks exist among professionals from other health care fields, such as medicine [34], nursing [35], or psychology [36]. Such approaches generally consider existing ethical guidelines developed by professional associations. For example, the American Medical Association has published an opinion that includes ethical guidelines intended for physicians when practicing telemedicine so that they comply with the code of medical ethics [37]. The similarities between the ethical framework proposed in this study and those American guidelines refer to ensuring objectivity and accuracy of website content; professional transparency and avoidance of conflicts of interest; best options in providing beneficial treatments to patients; safety, quality, and continuity of care; continuous protection of the integrity and security of patient information; proficient use of new technologies; understanding and overcoming their limitations to avoid harm. The differences in the American guidelines primarily refer to requiring another health professional to be present at the patient’s location to perform the necessary medical examinations, obtaining tailored informed consent for each feature of telemedicine, and ensuring that patients or their relatives understand the role of telemedicine technologies in providing the health care they need [37]. Such guidelines highlight that while embracing technological advancements, health care professionals, including pharmacists, must continue to uphold their ethical responsibilities.

By analyzing European pharmacists’ ethical codes and building an ethical framework for e-pharmacies, this study has successfully met its aim. This framework may be useful for developing codes of ethics for European pharmacists. The proposal of a common ethical framework for the conduct of pharmacists in e-pharmacies could be a starting point for reflection by professional associations of pharmacists and competent authorities in European countries, aiming to complement and enhance existing ethical guidelines. Such an ethical framework will also help pharmacists better understand and capitalize on the opportunities to contribute to patients’ health, ensuring they can fully leverage digital technologies to improve patient health. However, this framework is not exhaustive and offers a foundation for future research to expand beyond its limitations.

## Limitations

First, only e-pharmacy is addressed in this study and not telepharmacy, which does not refer to offering and selling medicines or other health products remotely but only to the remote provision of pharmaceutical consultations to the patient, which can involve the use of the internet and other communication channels, such as the telephone [97]. Second, only EU member states were investigated, as they have a common legal framework for selling medicines remotely via the internet [24], to evaluate the extent to which, operating within the same legal context, professional associations have adapted their codes of ethics to help pharmacists cope with the challenges of online activity with patients. Third, the codes of ethics and other relevant guiding documents for e-pharmacy ethics adopted by the professional associations of pharmacists or competent national authorities were researched without considering national-specific practices and interpretations or explanations for implementation if they were not published or mentioned on the researched websites or in the documents identified on these websites.

## Conclusions

When providing e-pharmacy services, pharmacists should observe professional ethics, apply specific rules of conduct, consider the legal context, and consider the benefits and risks associated with the online environment in which their professional relationship with patients is conducted. Within EU member states, codes of ethics and guidance documents from professional associations and regulatory bodies provide varied levels of regulation—from the absence of specific rules to detailed requirements regarding e-pharmacies—to protect patients’ rights in accessing medicines, health products, and related services. Pharmacists’ codes of ethics should keep pace with the development of e-pharmacies to ensure they remain relevant and effective in guiding ethical conduct and decision-making in the online environment.

To guide the ethical development of e-pharmacies, we propose an ethical framework for European pharmacists. This framework addresses ethical concerns grounded in the core principles of biomedical ethics: respect for autonomy, nonmaleficence, beneficence, and justice. The most important ethical issues resulting from this study include giving the choice of e-pharmacy, providing online professional transparency, operating robust websites, implementing strong privacy policies in e-pharmacy, avoiding data security breaches, providing personalized services to patients in e-pharmacies using synchronous communication, supporting the community, preventing digitally vulnerable patients’ exclusion from accessing e-pharmacies, and showing solidarity in the global e-pharmacy environment. This ethical framework will enable pharmacists to better understand and capitalize on the opportunities presented by advancements in digital technologies. It emphasizes its potential to enhance patient health by providing high-quality, safe, equitable, and reliable products and services through e-pharmacies, both locally and globally.

## Abbreviations

ABDA: Bundesvereinigung Deutscher Apothekerverbände (Federal Union of German Associations of Pharmacists)
AI: artificial intelligence
EAEP: European Association of e-Pharmacies
EHR: electronic health record
EU: European Union
FIP: International Pharmaceutical Federation
KNMP: Koninklijke Nederlandse Maatschappij ter bevordering der Pharmacie (Royal Dutch Pharmacists Association)
PGEU: Pharmaceutical Group of the European Union

## Data Availability

All data generated or analyzed during this study are included in this published paper.
